# Novel Vertical Localization Using Spectral Cues Analysis from Artificial Human Ears

**DOI:** 10.3390/s26175526

**Published:** 2026-08-31

**Authors:** Piyanat Sirisawat, Saiyan Saiyod, Woottichai Nonsakhoo

**Affiliations:** Hardware-Human Interface and Communications Laboratory (H2I-Comm Lab), Department of Computer Science, College of Computing, Khon Kaen University, Khon Kaen 40002, Thailand; piyanat_siri@cassia.kku.ac.th (P.S.); nonsakhoo@cassia.kku.ac.th (W.N.)

**Keywords:** sound source localization, vertical plane localization, time-domain analysis, signal analysis, direction of arrival estimation, acoustic signal processing

## Abstract

The ability to detect and localize the direction of the source of sounds has been with humans since ancient times, as humans can tell the direction of the sound coming from both the horizontal plane and vertical plane. Recent theory on why humans can distinguish the direction of the sound in the vertical plane is due to the difference between sounds reflected in human ears, more specifically, the pinna, which distort the sound in both the frequency domain and the time domain. In this paper, we will explore more on how to use the distortion in the time domain to localize the sound in the vertical plane. By analyzing the sound that is reflected in one of the artificial ears in the time domain, we use those data as a model to determine the sound coming from the vertical plane. The average angular error of the model is 4.86 degrees, with an RMSE of 6.90. The result confirms that we can analyze the sound in the time domain to determine the sound source direction in the vertical plane analyzed from only one microphone in the artificial ear.

## 1. Introduction

Sound Source Localization (SSL) is the ability to distinguish the direction or location of incoming sounds [1]. This system is most often used in robotics and underwater sound direction using sonar. The robot uses sound direction to determine its own position or recognizes the direction of incoming sounds and responds to them [2]. Underwater applications include locating the source of underwater sounds to investigate anomalies that may occur underwater, where cameras cannot provide clear visibility [3]. Currently, the use of SSL is being applied in a wider variety of applications, such as in Virtual Reality (VR) systems [4], for small robots [5], and even for handheld devices to help detecting and navigating for visually impair individual [6], making SSL much more useful. This is especially true in VR applications, where the ability to perceive sound direction can lead to the synthesis of personalized sound waves to imitate sounds coming from various directions [7]. Which in the nature, animals and even humans can perceive the sound direction using their ears. Humans can also acquire the ability to localize the sound source more accurately. With training and feedback to help localize the sound, the human subject can improve sound source localization greatly in both the horizontal and vertical planes [8].

SSL occurs in both humans and animals. Humans can distinguish incoming sounds in conjunction with their sight, while some animals rely primarily on sound direction for survival. For example, bats emit ultrasonic sound and perceive the direction of their surroundings by allowing sound reflections to determine distance and objects [9] SSL varies among animal species [10]. Some animals that rely more on sound direction have larger and more distinctive ears, allowing them to perceive and differentiate sounds better [11]. In humans, each has their own individual ear shape, which also reflects individual differences in SSL [12]. The changes in ear shape will disrupt the SSL in human subjects, but given time, they can relearn the SSL for the new ear shape [13]. There are two main directions in which the sound comes to the ears, the horizontal plane and the vertical plane. In normal hearing conditions, normal humans have a mean absolute error of 6.79 degrees in the vertical plane [14]. Spectral cues are changes in the sound wave caused by the head, torso, and pinna. Those changes depend on the direction of the sound because the changes are separate for each ear. They can localize the sound by using only one ear. There is an experiment on sound levels and the ability to localize sound, especially in people with hearing loss in one ear. They conclude that a low sound level will decrease the ability to localize sound. They will need an optimal sound level to help the patient localize the sound using one ear [15]. Spectral cues are important in determining the sound source direction in the vertical plane. Analyzing those changes should help us determine the sound source direction in the vertical plane using one ear.

Early experiments on SSL involved placing microphones in the artificial human ear to detect differences in sound received from different directions [12]. It was discovered that the sound received from the left and right ears differed in loudness and time difference of arrival (TDOA). This led to the development of two theories on sound direction perception. The first is based on the difference in sound intensity of the two ears (Interaural Intensity Difference: IID), and the second is directionality based on the difference in TDOA of the two ears (Interaural Time Difference: ITD) [16]. Both methods use two microphones to determine sound direction. In terms of loudness, a sound source closer to one microphone will have a louder sound than the other, allowing for the perception of the sound’s direction. Similarly, in ITD, the microphone closer to the sound source will pick up the sound before the microphone that is further away [17].

However, localizing the sound direction using only two microphones has limitations. The system can only perceive sound in the direction that it is facing [18]. This introduces the cone of confusion, which the system cannot determine if the sound is coming from the front or the back. This means the whole setup must move to face another direction for the system to correctly determine the SSL. Thus newer setup has to use two more microphones, totaling four microphones in a microphone array setup [19,20]. This eliminates the cone of confusion and allows for 360-degree sound localization in the horizontal plane. Increasing the number of microphones in the system further will increase accuracy [21], at the cost of increased computational power.

The microphone array method for SSL remains fundamental for robotics. As robots do not require highly precise directional accuracy. Because they have cameras to help locate the sound source direction [5,18,22]. Currently, in addition to locating the sound source or speaker direction, more studies are developing a system to pinpoint the origination of the sound within a picture [23]. The number of microphones in the array must be increased to achieve much more accurate sound direction and locate the sound source position in the visual cue [24]. This comes at the cost of more extensive sound analysis. Instead of comparing just one or two pairs of microphones, four or more pairs are needed to improve accuracy and enable 3D sound direction detection [25]. The microphone array can be distributed to reduce the number of the microphones in the array. Instead of placing them next to each other, they can be placed in a 3D distributed space to help reduce the computational cost [26], when compared to humans or animals, which use only two ears, but can still locate sound direction in both thehorizontal and vertical directions, in 3D. The difference between a normal microphone and the human ear lies in the presence of the outer ear, the pinna [12].

We know that changes in the shape of the human ear affect its perception of sound direction [13]. Therefore, it can be said that the most important organ for human sound localization is the pinna. Thus, if we were to add a human ear shape to a microphone, we might be able to locate sound direction in both the horizontal and vertical planes, just like with the human ear, which uses only 2 ears and can locate sound direction in 3D. However, simply adding a human ear is not enough to localize the sound direction in the 3D plane. An accurate analysis technique is required. Neither the IID nor the ITD analysis can detect the patterns of sound reflections in the pinna. We know that sound reflecting in the pinna alters its characteristics [27]. Therefore, a proper analysis technique is needed to detect these differences, specifically, frequency analysis. This means changing the sound wave from the time domain into the frequency domain. These frequency changes follow a pattern based on the pinna’s contours even with an artificial one [28,29]. The change in frequency patterns varies depending on the direction of sound due to different direction in which sound impacts the pinna’s contours. These differences can be used to determine vertical sound direction, along with IID/ITD for horizontal direction [30]. Furthermore, one study uses differences in sound before and after entering the ear-shaped microphone. This allows for more accurate sound direction determination by reducing noise and focusing only on the sound patterns impacting the ear [31]. We call the technique that uses the human ear shape and the analysis of the frequency change the Head-Related Transfer Function (HRTF).

Although HRTFs can be used to localize the sound direction in horizontal, vertical, and even 3D directions, the limitation of HRTFs is the need to transform audio data into frequency patterns, which will allow the comparison of frequency changes from the sound reflecting the pinna or other parts of the body [32]. This means that HRTFs need precise frequency characteristic as different sounds reflected from difference object would also have different frequencies. Thus, the frequency change from the reflection in the pinna will also differ for each sound [33]. The HRTFs will need to record multiple frequency patterns to effectively localize each sound source. This makes the required dataset and SSL computationally complex. HRTFs are often used in conjunction with Machine Learning (ML) or Convolutional Neural Networks (CNN) to help with sound localization much more accurately [34].

From all the methods mentioned above, the IID and ITD can be used in horizontal plane sound localization. However, those methods have a cone of confusion. The microphone array is needed to help differentiate sounds in front/behind the system and eliminate this cone of confusion and to localize the vertical sound direction requires a microphone array with a specific configuration. In addition, for greater accuracy, a larger number of microphones in the array is needed [25]. This will make the system computationally more complex. However, new research suggests that the accuracy of microphone arrays can be improved even with a reduced number of microphones in the array by arranging in a specific layout [35]. But if we want to reduce the number of microphones, we need to use extra equipment, devices shaped like human or animal ears. These can help the system with the localization of sound entering the microphones by analyzing the changes in sound frequency that reflect from the pinna using HRTFs. The changes in frequency resulting from sound impact in the ear allow for SSL. Normally, either ML or a CNN is used to help in decision-making, resulting in higher accuracy. However, this comes at the cost of more complex and time-consuming calculations.

This leads to our approach for analyzing sound direction using a human ear-shaped device in conjunction with a microphone. The analysis will be conducted in the time domain, without relying on frequency differences. We know that the sound waves reflecting in the pinna will have a change in frequency. They also travel an extra distance compared to sound that directly enters the ear. This results in a time delay between those two sounds. If we can identify the difference in those delays from multiple directions, we can use those differences to localize the sound in the vertical plane without needing a microphone array or converting the sound from the time domain to the frequency domain.

## 2. Materials and Methods

**Figure 1:**
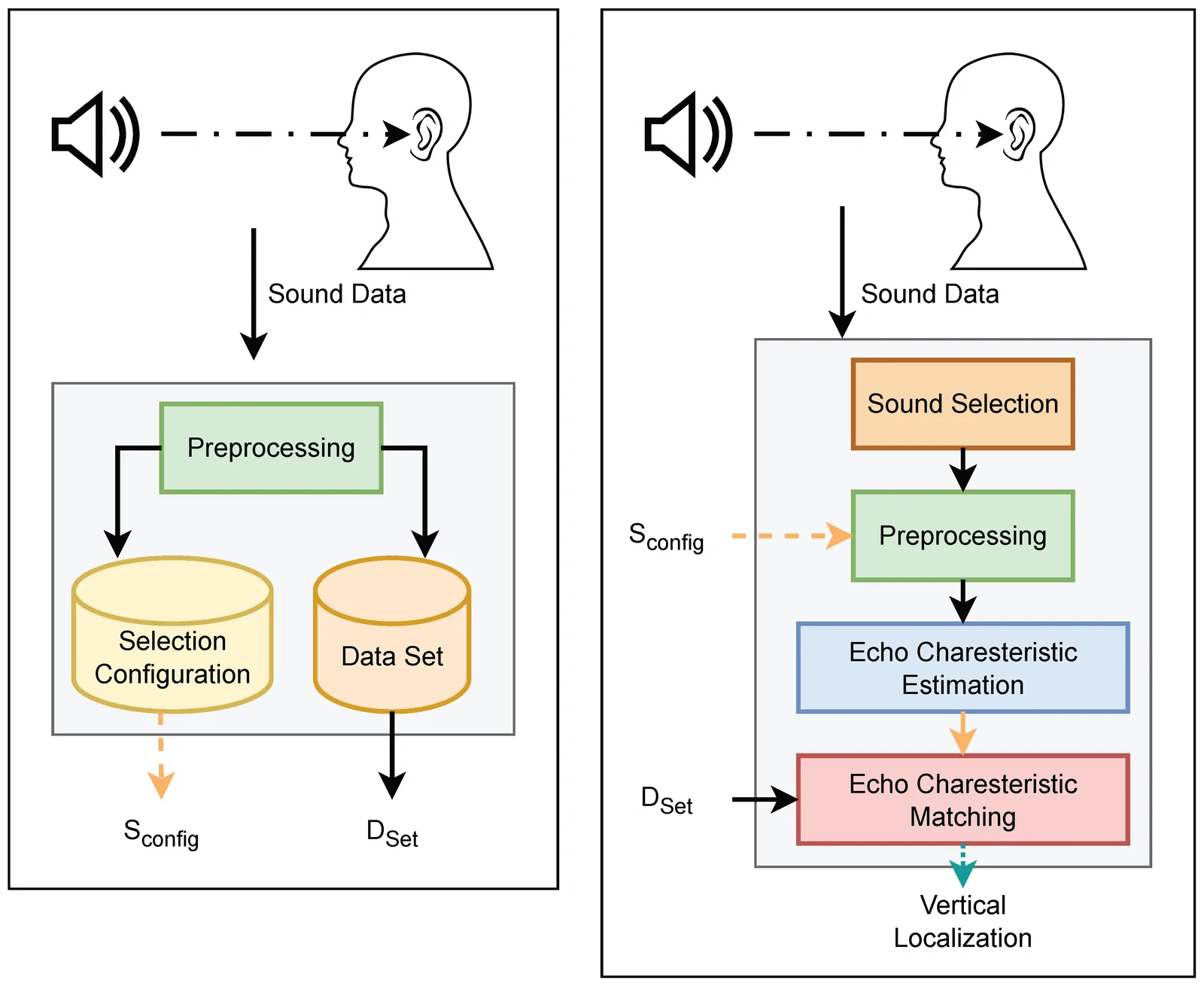
The vertical localization system in our research.

To localize the sound source direction, it is necessary to create a dataset specific to this experiment. The same equipment will be used to create each dataset. Once the dataset is obtained, we will find an appropriate starting position for the sound and the optimal window size to minimize errors from analyzing irrelevant data. After analyzing the sound, we can use the sound from this dataset to find the similarity with an unknown sound direction. The overall system is shown in Figure 1.

### 2.1. Sound Wave

A sound wave is a phenomenon where the transmission medium vibrates and transmits those vibrations through itself. The transmission medium can be a gas, solid, or liquid. In a physical system, the sound wave (s(t)) is normally represented by Equation (1).

(1) s(t)=Acos(ωt+ϕ)

where ω represents angular frequency, which is the number of radians per unit of time, normally referred to as repetitions of an event per unit of time, or ω=2πf, commonly known as the Hertz (Hz). The Hertz represents one cycle per second and is normally used as a natural unit of frequency. Humans normally can hear the sound wave at a certain frequency, between 20 Hz to 20 kHz in normal atmospheric pressure. Frequencies higher than those audible to humans are called ultrasonic. However, in this research, we will focus on human speech, which falls within the frequency range that humans can perceive.

All recorded samples and all subsequent correlation operations in this study are real-valued. Equation (1) is used only to introduce the amplitude, angular frequency, and phase of a real sinusoidal component; the proposed localization algorithm operates directly on sampled waveforms and does not introduce a complex-valued signal representation.

### 2.2. Pinna Multipath

**Figure 2:**
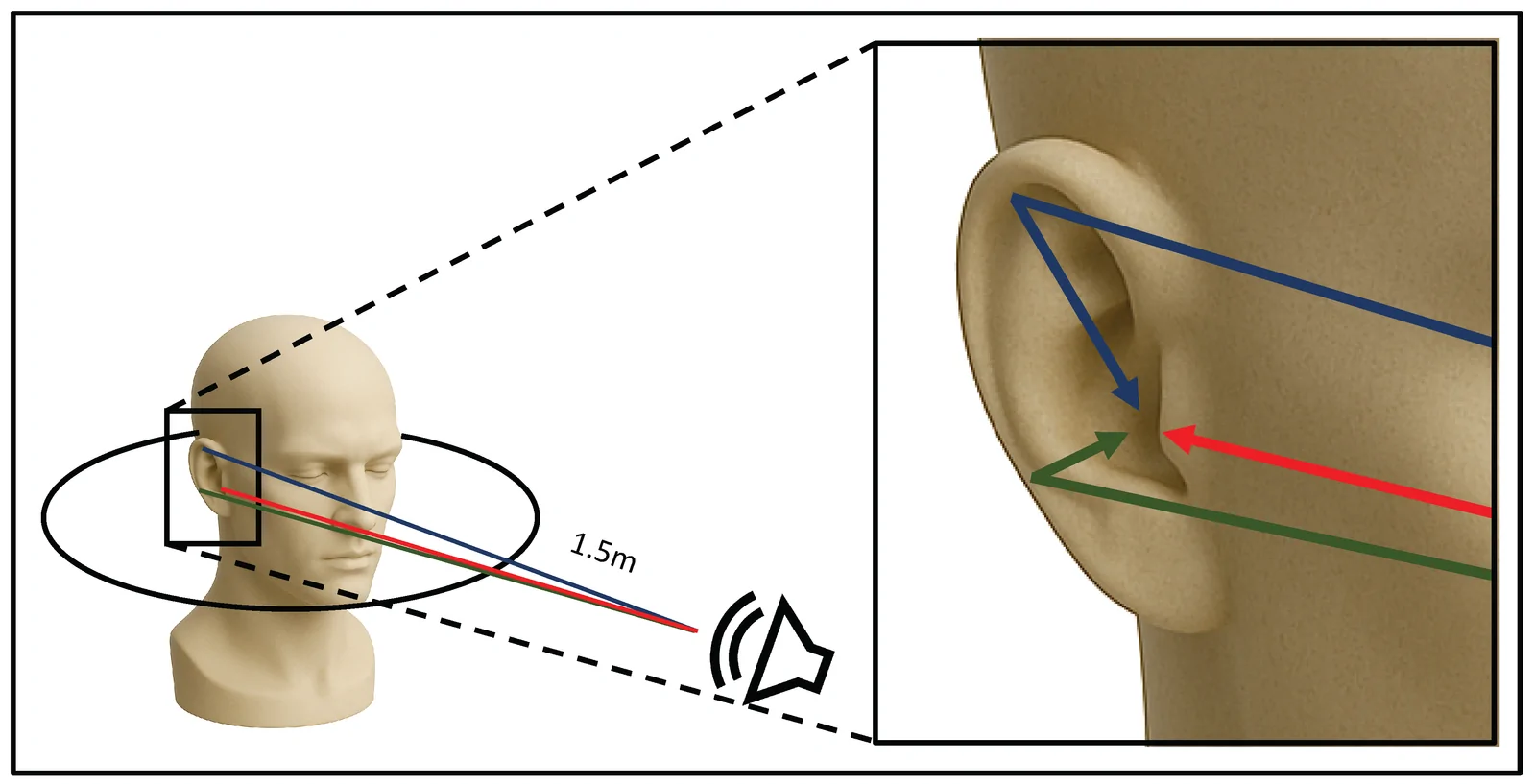
Sound arrives at and is reflected in the ear.

Sound multipath is a phenomenon that occurs when a sound wave scatters when traveling to the receiver or listener. This phenomenon can occur when the sound wave reflects upon hitting any surface, such as floors, walls, ceilings, or any other material, including human ears. The pinna multipath refers to the phenomenon where the sound wave is reflected in the ear Pinna. The scattered sound will come to the receiver or listener at different times, normally referred to as echoes. The sound wave that takes the direct path will arrive first, and other sound waves that are reflected on other surfaces will travel further and thus take more time to arrive at the receiver or listener. Due to the unique shape and contours of the human ear, sound waves traveling from different directions will be reflected on these shapes and contours at different locations. This causes the sound waves to take different amounts of time to enter the ear canal from each vertical direction, as shown in Equation (2) and Figure 2. Also, sound waves coming from different vertical angles will have different reflection locations within the pinna. Therefore, we can use these time differences to determine the direction of sound in the vertical plane by analyzing the sound in the time domain.

Let x[n] denote the sampled source waveform and let $z_{\theta }\left[n\right]$ denote the signal recorded at vertical incidence angle θ. A discrete multipath model for the received signal is

(2) $$z_{\theta }\left[n\right]=\sum_{k=0}^{K_{\theta }-1}a_{k}\left(\theta \right)x\left[n-d_{k}\left(\theta \right)\right]+\eta \left[n\right],$$

where $K_{\theta }$ is the conceptual number of resolvable propagation contributions, $a_{k}\left(\theta \right)$ is the effective gain of contribution *k*, $d_{k}\left(\theta \right)$ is its nonnegative integer delay in samples, and η[n] represents background noise and unmodeled effects. The direct contribution is indexed by k=0 and is aligned so that $d_{0}\left(\theta \right)=0$; terms with k≥1 represent delayed reflected or scattered contributions. The corresponding delay in seconds is $\tau_{k}\left(\theta \right)=d_{k}\left(\theta \right)/f_{s}$, where $f_{s}$ is the sampling rate.

The angle-dependent discrete impulse response associated with Equation (2) is

(3) $$h_{\theta }\left[n\right]=\sum_{k=0}^{K_{\theta }-1}a_{k}\left(\theta \right)\delta \left[n-d_{k}\left(\theta \right)\right],$$

so that the same model can be written compactly as

(4) $$z_{\theta }\left[n\right]=\left(x*h_{\theta }\right)\left[n\right]+\eta \left[n\right].$$

The scalar $a_{k}\left(\theta \right)$ is an effective broadband path gain and is allowed to vary with incidence angle. A more detailed frequency-selective model would replace each scalar gain by a path filter; however, the present time-domain implementation does not identify such filters. More importantly, the localization algorithm does not estimate the individual physical values $a_{k}\left(\theta \right)$ and $d_{k}\left(\theta \right)$. They motivate the angle-dependent multipath model, while the implemented method uses a normalized lagged self-correlation profile as an empirical signature of the combined contributions.

**Figure 3:**
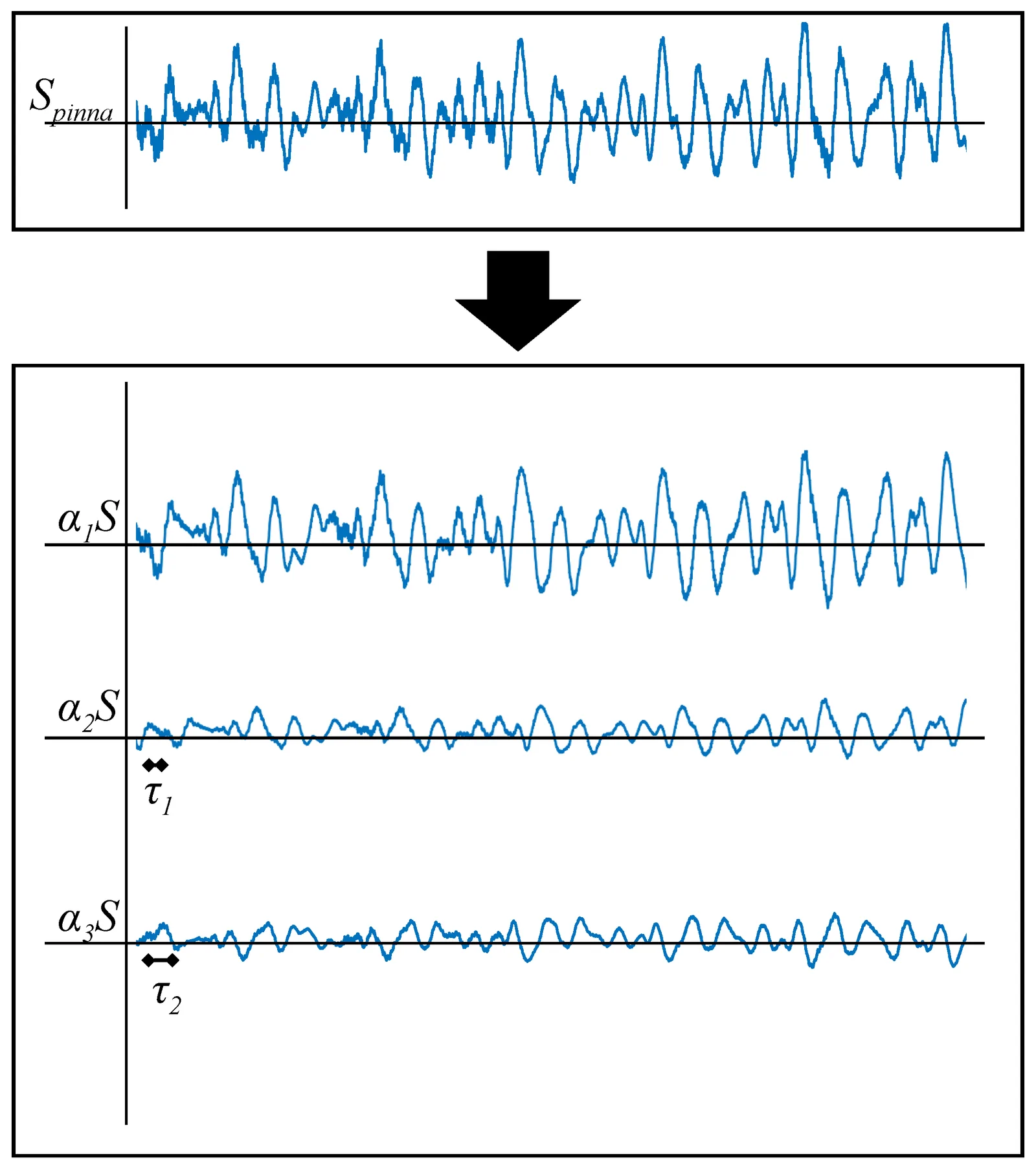
The example of a sound wave in a pinna-multipath.

Figure 3 illustrates a direct path and two reflected paths only to explain the multipath concept. The actual classifier neither fixes $K_{\theta }$ to three nor selects a prescribed number of reflection peaks. Instead, it retains a vector of lagged self-correlation values over a fixed lag interval, as defined in Section 2.4.

**Figure 4:**
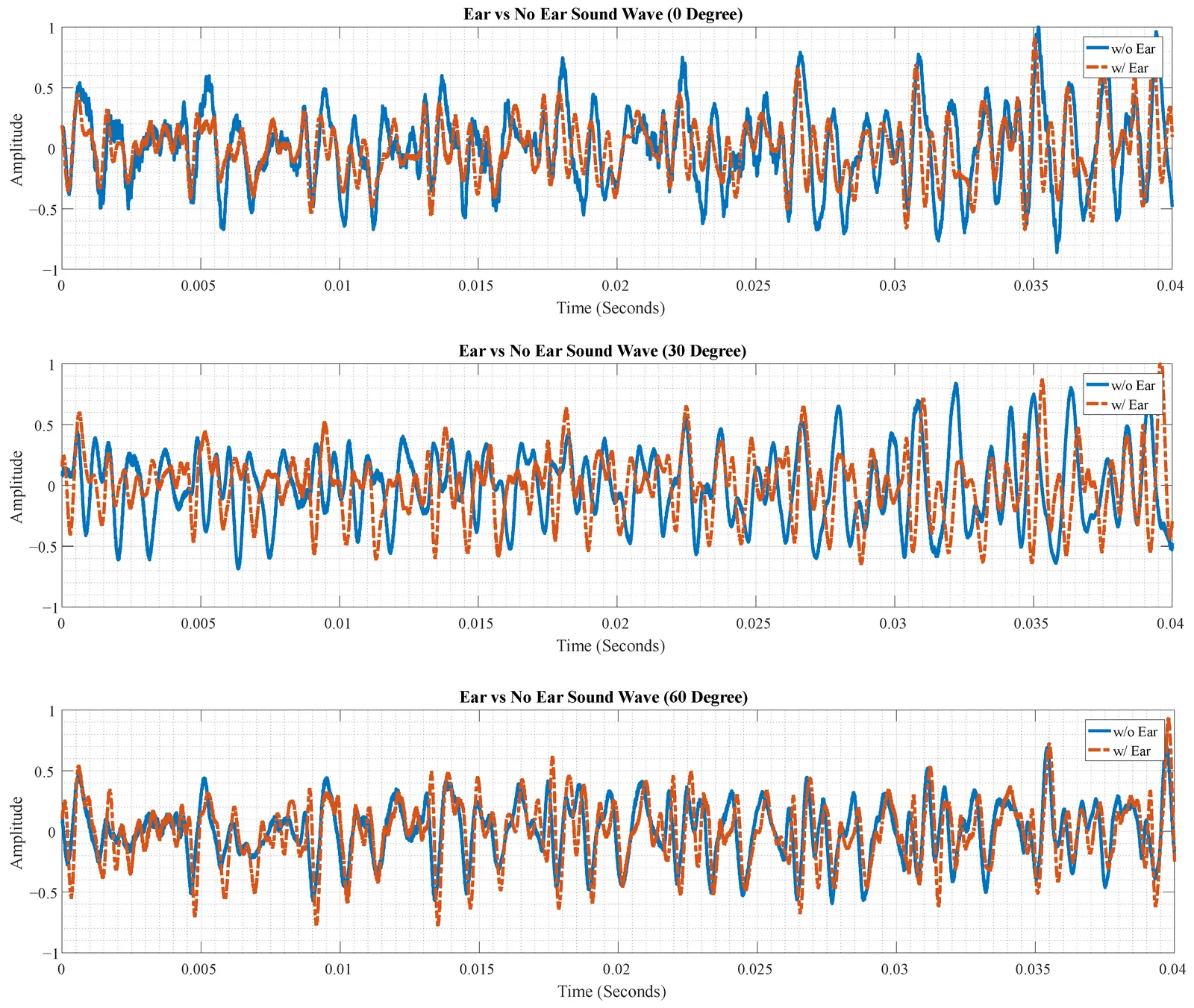
Comparison of a sound wave with an artificial ear and without an artificial ear.

Sound waves that are reflected in the outer ear will be a different waveform from sound waves that do not reflect in the outer ear. First, we record the sound entering the microphone without the outer ear. We try to keep all other unrelated variables as close to each other as possible. Then, we record the sound again with an artificial ear placed over the microphone to compare the waveform characteristics. The comparison of sound wave characteristics without the artificial ear and sound entering and reflected in the artificial ear is shown in Figure 4.

**Figure 5:**
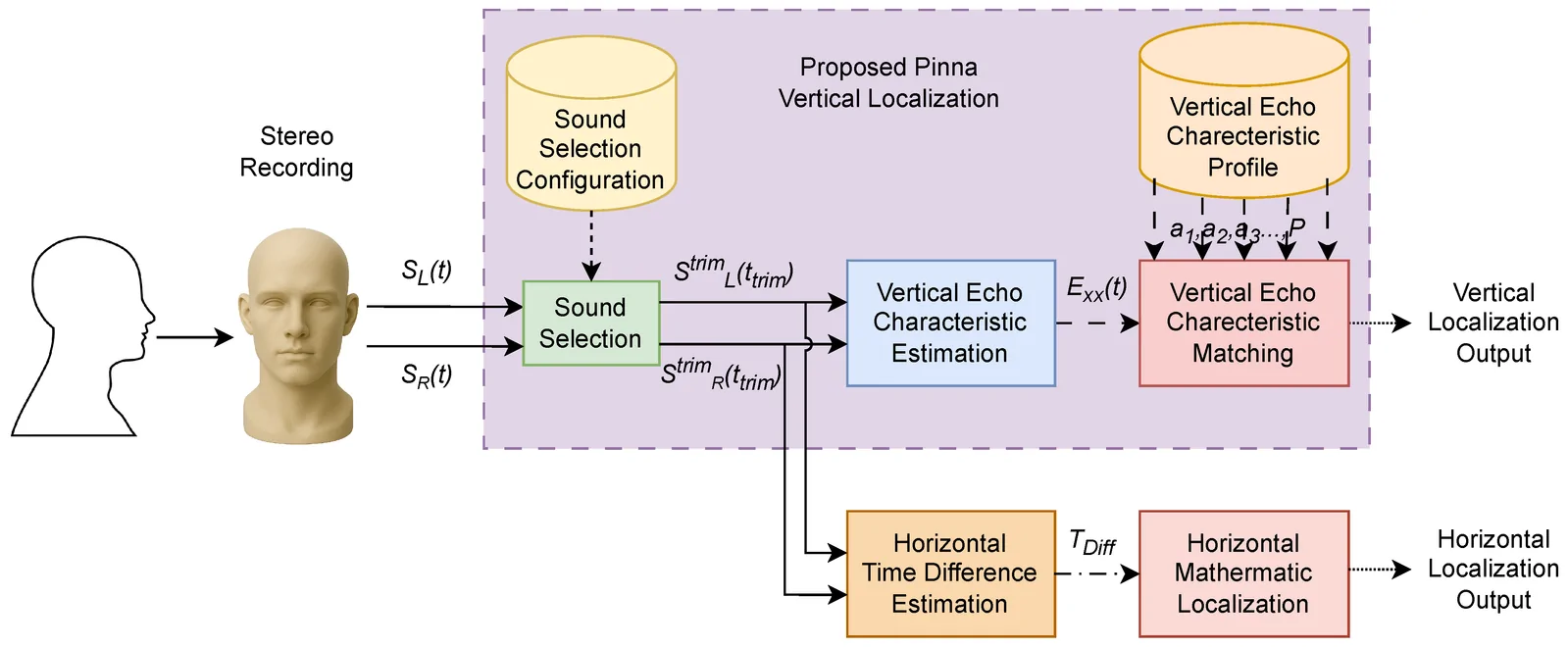
Proposed system for vertical localization.

Our system will use an artificial ear with the microphone to record the sound. Then select the appropriate sample and trim the sound so we can use it to find the pinna-multipath of the sound wave. By using the Echo Characteristic Profile (ECP) that we made beforehand, we can use it to localize the sound in the vertical plane, which results in the sound direction in the vertical plane. The system is shown in Figure 5.

### 2.3. Sample Collection

**Figure 6:**
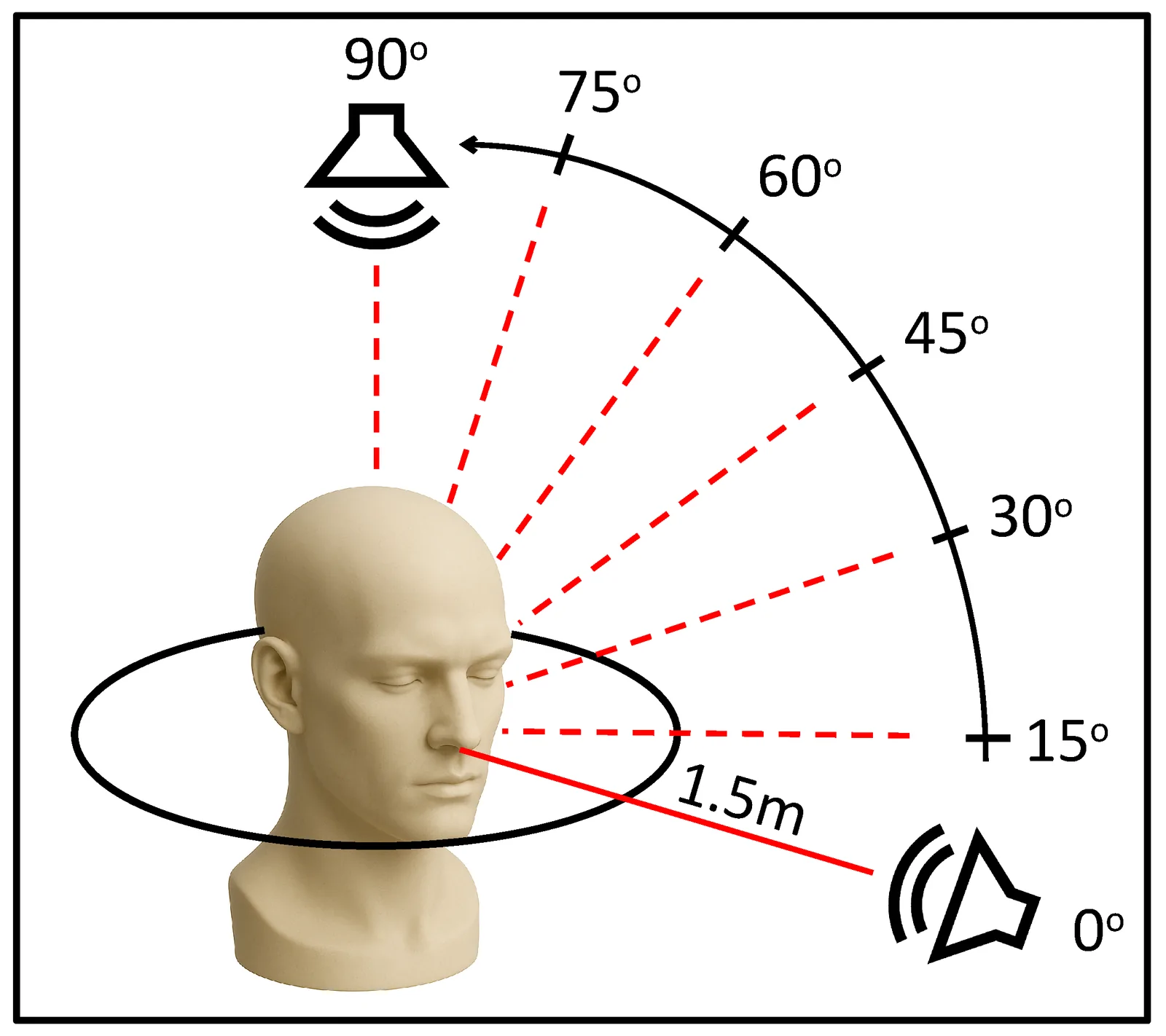
The angle for recording the sound in the vertical plane.

The sound sample is the synthesis of a human sound saying “hello” using Google Translate. The sound will be collected using a microphone in an artificial human ear. We will also be using a spherical ball to replace the human head to closely resemble the human anatomy. The size of the ears and ball is twice the size of the ears and head of a normal human. The length of the ear is 12 cm and the distance between the ears is 30 cm. This will help us analyze the sound reflection in the human ear more clearly. The sound sample will be recorded using a sound resolution of 192 kbps with an External Sound Card model ASUS Xonar U5. We also collect an additional sound sample with a lower sound resolution of 48 kbps to compare with a higher sound resolution. The height is 1 m from the floor, and the distance between the speakers and the ball is 1.5 m. The sound samples are collected at a vertical angle at an angle of 0 degrees and increasing in increments of 15 degrees until reaching an angle of 90 degrees or above the ball, as shown in Figure 6.

#### 2.3.1. Sound Trimming

In order to analyze the sound wave collected from the artificial human ear, we need a suitable window size and data range. In this study, we used MATLAB 2016a to modify and analyze the sound wave. If the window size is too large, it will need more computing power to calculate the data. But if the window size is too small, the essential data for the vertical localization might not be there. This also applies to the data range. If the data range is outside the essential data for vertical localization, the analysis result might not be able to localize the sound direction. In this step, we will also select one of the sounds from the left or right ear. We will use the sound with higher energy to provide better accuracy in sound localization.

#### 2.3.2. Data Range Configuration

Other than the window size, the appropriate data range or the location of the sample that we need to place the window size on is also important because we will need the sound to have both the main sound and the reflected sound, which will help us analyze the echo.

**Figure 7:**
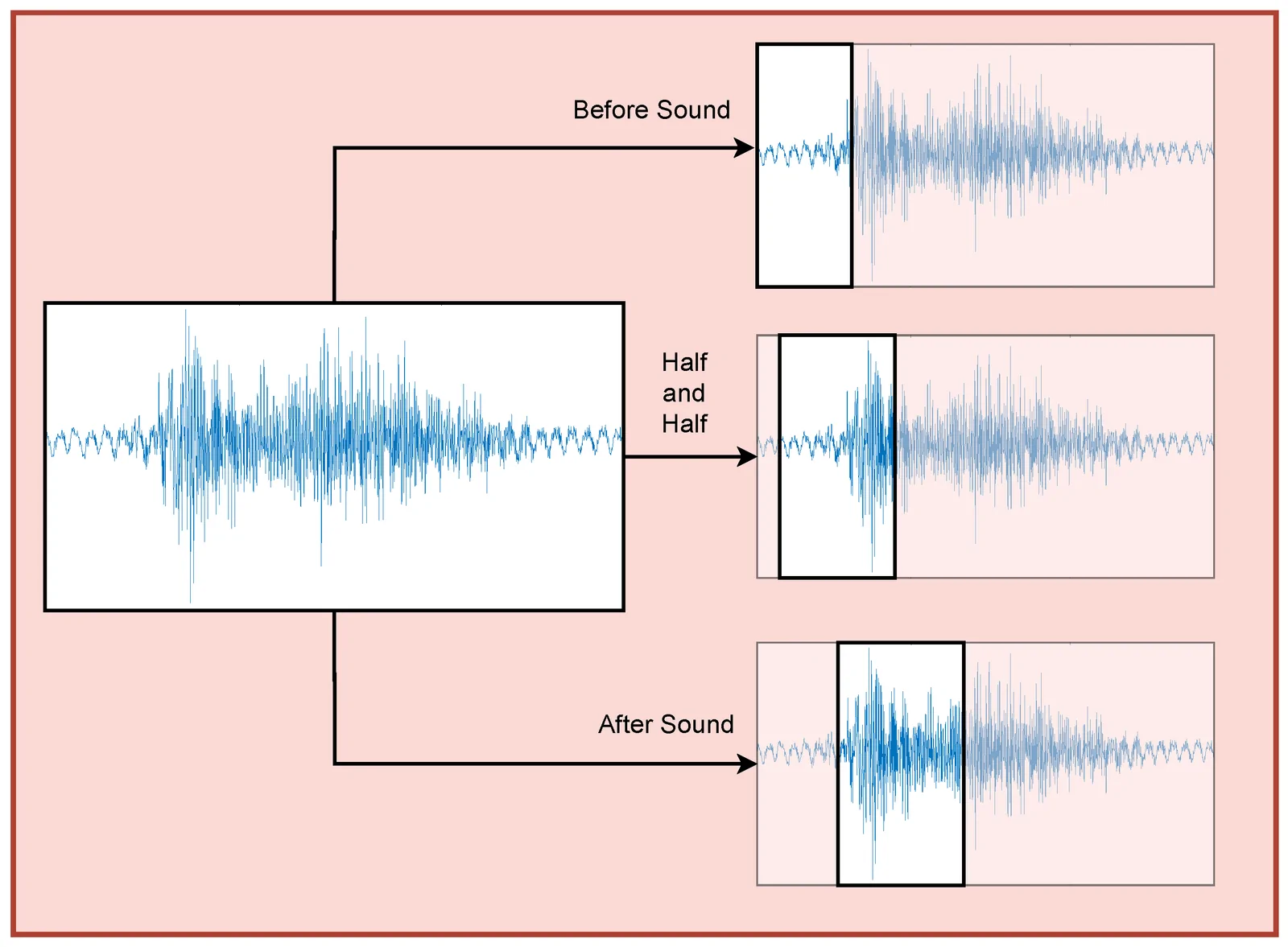
Example of the data range used in the experiment.

There are three start locations that will be tested. The first is before the main sound comes to the ear. This location will mostly have the sound before the sound and have only a little bit of the sound coming into the ear. The second location is half of the sound. In this location half of the window size will be the sound, and half of it is before the sound coming to the ear. The last location is the full sound. In this location, the window size will sit in the sound itself will a little bit sitting before the sound coming into the ear, as shown in Figure 7.

In the result from each of the data range, the location before the sound has the lowest echo sample because it only has a little of the sound that we are interested in analyzing (refer to Echo Characteristic Profiles method in Section 2.4), while the other two, one where there is half no sound and half sound and the full sound, have almost the same result. But the full sound will sometimes miss the start of the sound. So the most appropriate data range is half no sound and half sound, or half and half. This will guarantee that the start of the sound or the main sound is intact and can be used to find the Echo characteristic from the pinna multipath.

#### 2.3.3. Window Size Configuration

**Figure 8:**
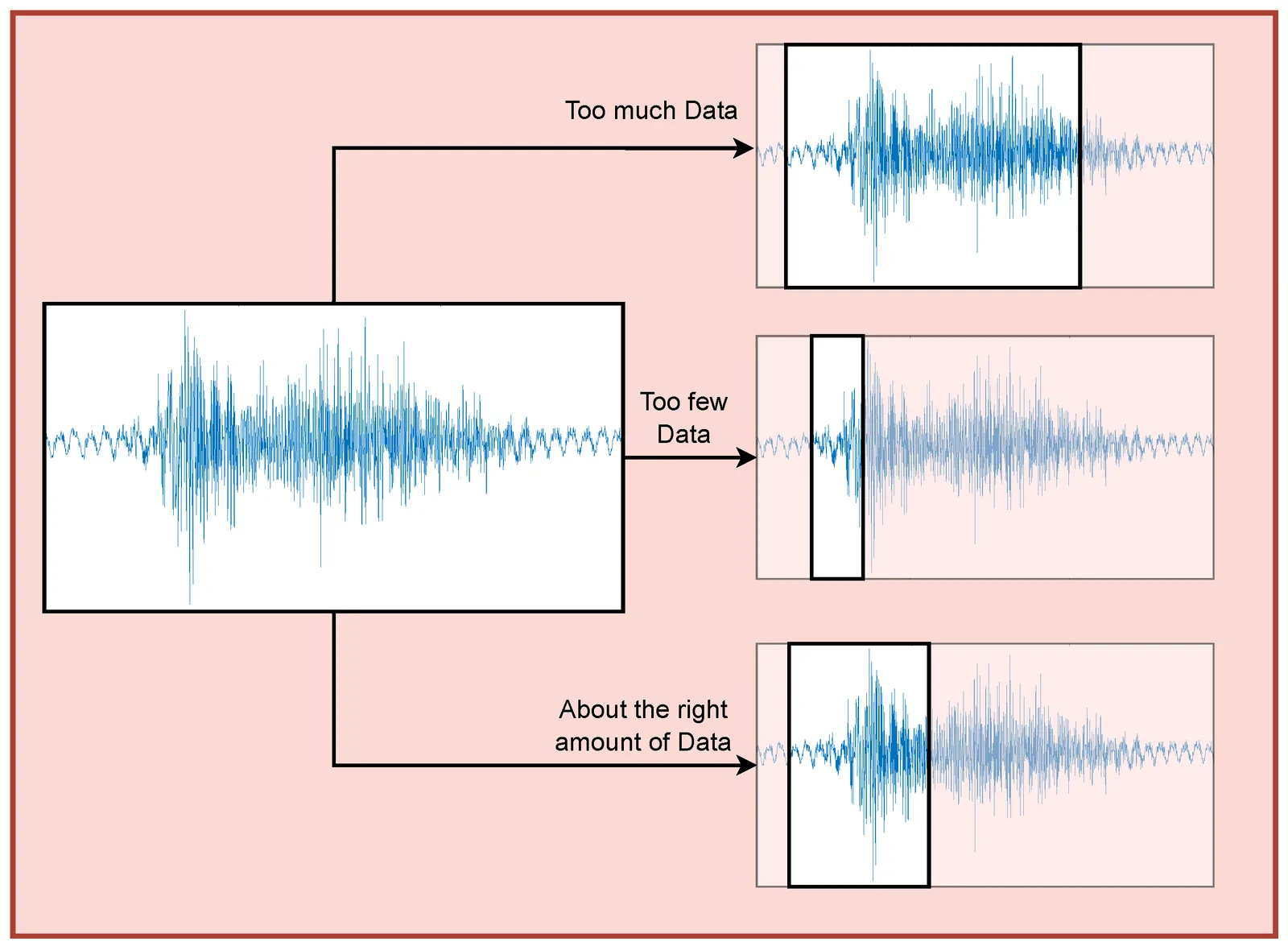
The window size used in the experiment covers the essential data.

A suitable window size is needed to help localize the sound in the vertical plane much more efficiently. Because the sampling rate of the sound that we recorded is 192 kbps, we would need an appropriate window size because a small window size will not be able to detect echo, while a larger window size would need more computation resources, as shown in Figure 8.

The window size that we will be testing is from 100 to 48k window and finds an appropriate windows size that can analyze echo. We discovered that a small window size of 100 to 6000 cannot determine the precise echo characteristic, while the large window size (48,000) is not different from the smaller window size. From the result, we found that the appropriate window size is 24,000 or 125 ms. It is not too small and can determine the echo characteristic without using much computing resources.

As for the sound with a sampling rate of 48 kbps, we will be using the same configuration as the sound with a sampling rate of 192 kbps, but it will have a lower window size of 6000 to match the 125 ms that we use at the sampling rate of 192 kbps.

### 2.4. Echo Characteristic Profiles

The combined direct, reflected, and scattered contributions produce angle-dependent correlation patterns. We represent these patterns by an Echo Characteristic Profile (ECP). For a window containing *N* recorded samples, the normalized nonnegative-lag ECP is defined as

(5) $$e_{\theta }\left[l\right]=\frac{\sum_{n=l}^{N-1}z_{\theta }\left[n\right]z_{\theta }\left[n-l\right]}{\sum_{n=0}^{N-1}z_{\theta }^{2}\left[n\right]},\;l=0,\ldots ,L-1,$$

where *ℓ* is a lag in samples and *L* is the number of retained nonnegative lags. The lag time is $\tau_{l}=l/f_{s}$. The implementation uses a window of N=24,000 samples, computes the normalized correlation of the signal with its lagged copy, discards negative lags, and retains the first L=900 values. We refer to this operation as normalized lagged self-correlation. Thus, the ordinate $e_{\theta }\left[l\right]$ is a normalized correlation coefficient rather than a calibrated physical attenuation coefficient. Profiles obtained from the calibration recordings are stored as angle-dependent templates for later comparison.

The mathematical connection between the multipath model and the ECP can be seen by expanding the idealized lagged self-correlation of Equation (2). If the source and noise are uncorrelated, then

(6) $$r_{z_{\theta }}\left[l\right]=\sum_{i}\sum_{j}a_{i}\left(\theta \right)a_{j}\left(\theta \right)r_{x}\left[l+d_{j}\left(\theta \right)-d_{i}\left(\theta \right)\right]+r_{\eta }\left[l\right].$$

**Figure 9:**
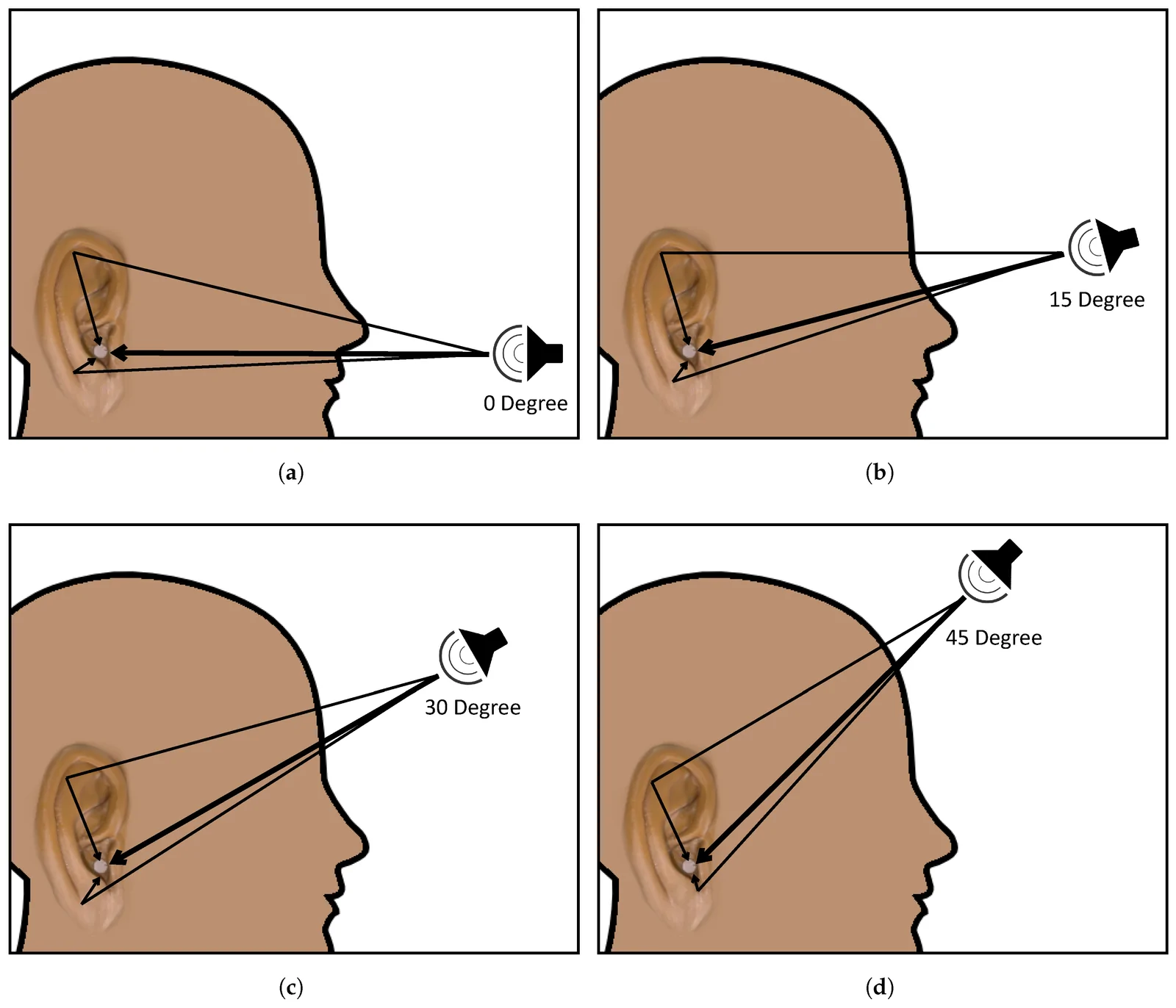
The vertical angle used in this experiment and the example of the reflected sound in the pinna by estimation: (**a**) 0 degrees, (**b**) 15 degrees, (**c**) 30 degrees, and (**d**) 45 degrees.

Equation (6) shows that the self-correlation features are associated with pairwise path-delay differences, $d_{i}\left(\theta \right)-d_{j}\left(\theta \right)$, and are shaped by the source self-correlation $r_{x}\left[l\right]$. Consequently, there is generally no one-to-one correspondence between an ECP peak and one physical pinna reflection. Peaks may overlap, may be broadened by the source waveform, or may be affected by noise and the finite observation window. For this reason, the localization method compares the complete *L*-sample ECP rather than attempting to recover a fixed number of reflection paths. Figure 9 illustrates how the resulting profiles vary with angle.

**Figure 10:**
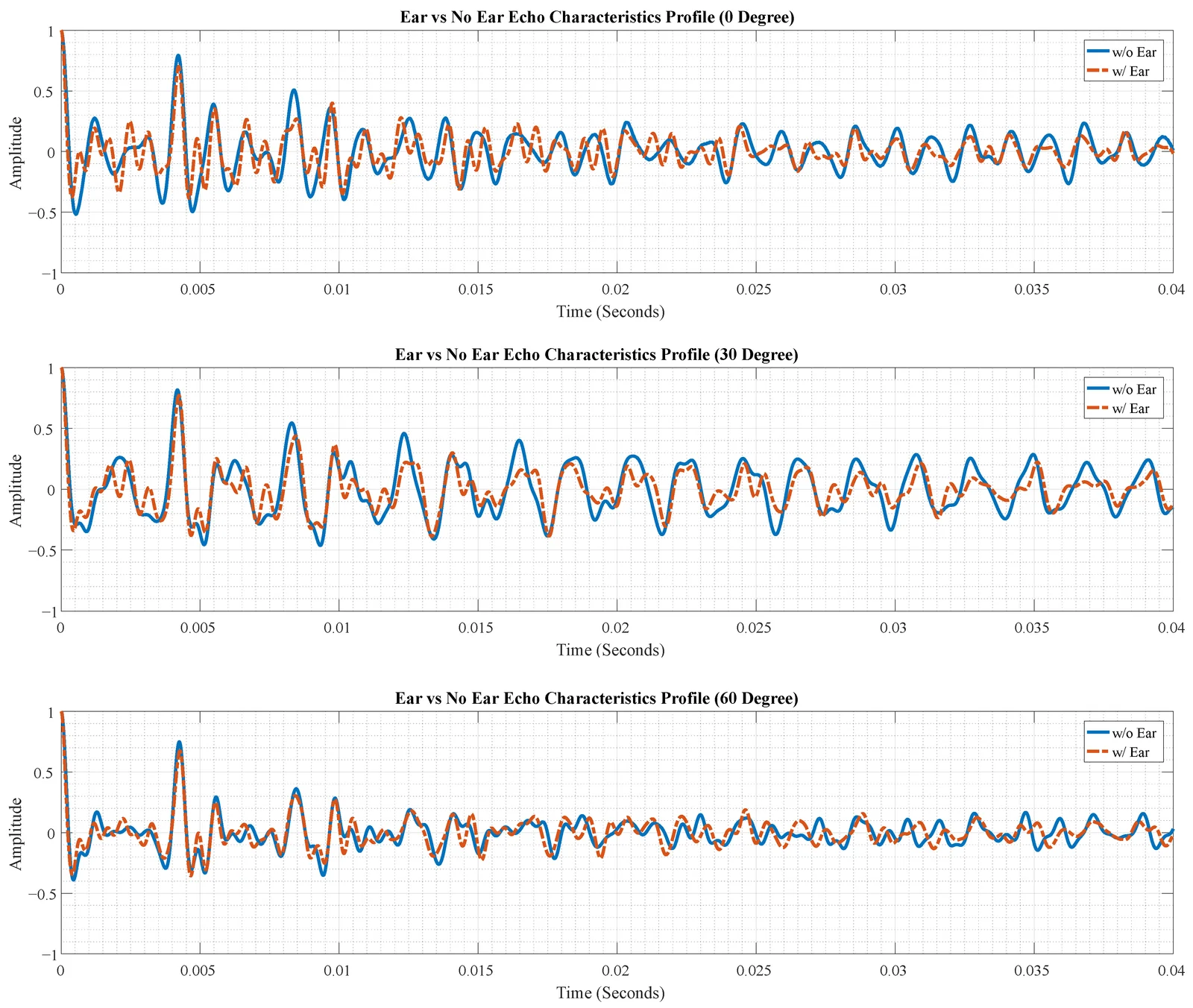
Comparison of the Echo Characteristic Profile of the sound with and without an ear.

**Figure 11:**
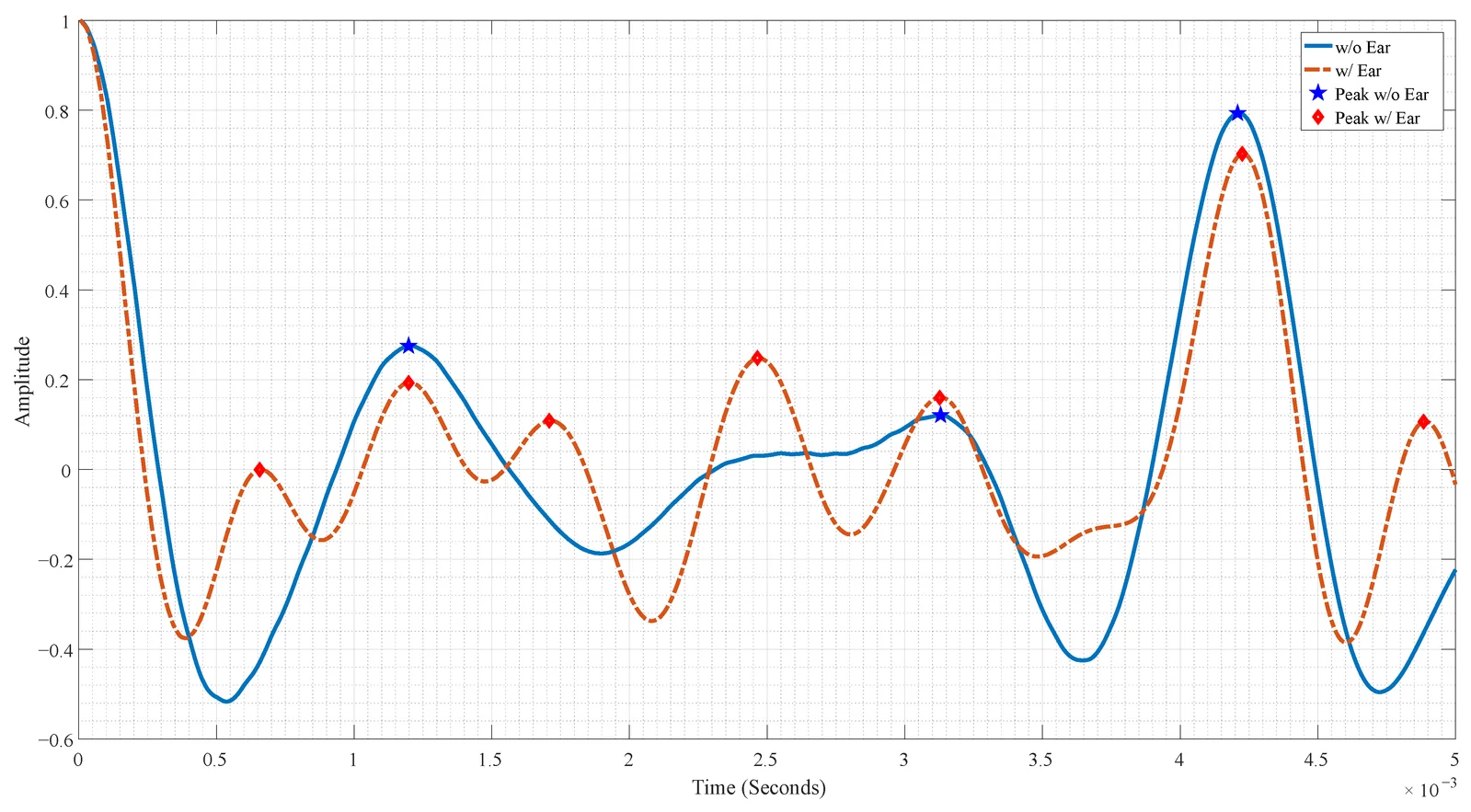
The peak-count on the Echo Characteristic Profile of the sound with and without an artificial ear at 0 degrees.

The signals recorded with and without the artificial ear produce visibly different normalized lagged self-correlation profiles. The artificial ear changes the correlation structure over nonzero lags because the recorded waveform contains the combined direct and pinna-mediated contributions, as shown in Figure 10. A quantitative peak-count analysis of this difference is treated separately from the mathematical definition of the ECP. These are shown in Figure 11, in the time window of 0.005 s (5 ms).

We are using the same sound when recording those two sounds from each angle. The sound without an artificial ear will be used to provide a baseline of the sound that reflected from the environment, head included. When we compare it to the sound that we recorded with an artificial ear, they show significantly different peak sound energy on the graph. The only difference is the presence of the artificial ear. We can assume that the difference in those Echo Characteristic Profiles is due to the artificial ear alone.

**Figure 12:**
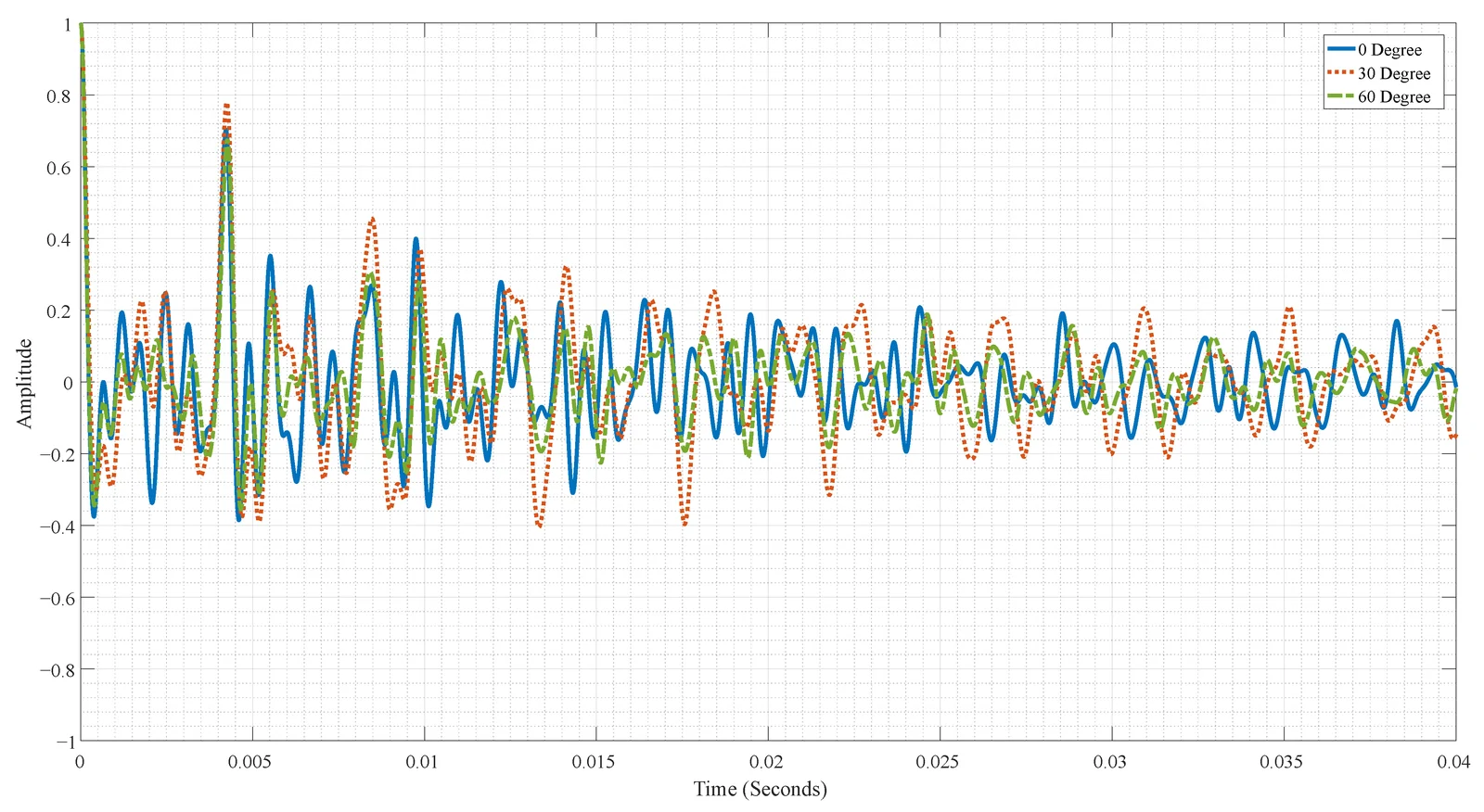
Comparison of the Echo Characteristic Profile of the sound with an ear at different vertical angles.

In Figure 12, the different of the Echo Characteristic Profiles from each of the vertical angles show significant differences in both the energy and each of the delay differences. This means that we can use this data to determine the sound source direction in the vertical plane.

### 2.5. Time-Delay Localization Method

In this experiment, we aim to determine the sound source direction in the vertical plane using only one microphone with an artificial ear. We will analyze the sound in the time domain, focusing on the reflections of the sound in the pinna. We have confirmed that the sound reflections in the pinna of the human ear can pinpoint the direction of sound from various directions, both vertically and horizontally, all while using only one ear. Previous experiments show that changes in the ear structure have an effect on the human ability to determine the sound direction. Therefore, we can conclude that the ear pinna is a crucial organ for sound direction in humans. In order to mimic human hearing accurately, the use of the human pinna is crucial. The most important aspect is understanding how the human ear affects the sound waves is reflected in the pinna and the ability to determine their direction. We know that the human ear has a unique shape that reflects sound differently when the sound is coming from different angles in the vertical plane. This results in distinct characteristics for each sound direction in each of the vertical directions. Therefore, analyzing these vertical sound differences will focus on analyzing these reflections, as shown in our previous Echo Characteristic Profiles dataset.

The path gains and delays in Equation (2) explain why recordings from different incidence angles can produce different ECPs. They are not separately calibrated by the implemented classifier. Instead, the calibration stage records repeated sounds at known vertical angles, calculates their normalized ECPs using Equation (5), and stores representative profiles. This empirical calibration incorporates the combined effects of path gains, pairwise delay differences, the artificial-ear geometry, and the recording chain.

Let e[l] be the ECP of a test recording and let $p_{j}\left[l\right]$ be the stored ECP for angle class *j*. The implementation computes their normalized cross-correlation:

(7) $$\rho_{j}\left[q\right]=\frac{\sum_{l}e\left[l\right]p_{j}\left[l-q\right]}{\sqrt{\sum_{l}e^{2}\left[l\right]}\sqrt{\sum_{l}p_{j}^{2}\left[l\right]}},\;S_{j}=\max_{q}\rho_{j}\left[q\right],$$

where *q* is a relative shift between the two length-*L* profiles and $S_{j}$ is the maximum normalized cross-correlation coefficient. The best-matching stored profile is

(8) $$j^{★}=\arg\;\max_{j}S_{j}.$$

**Figure 13:**
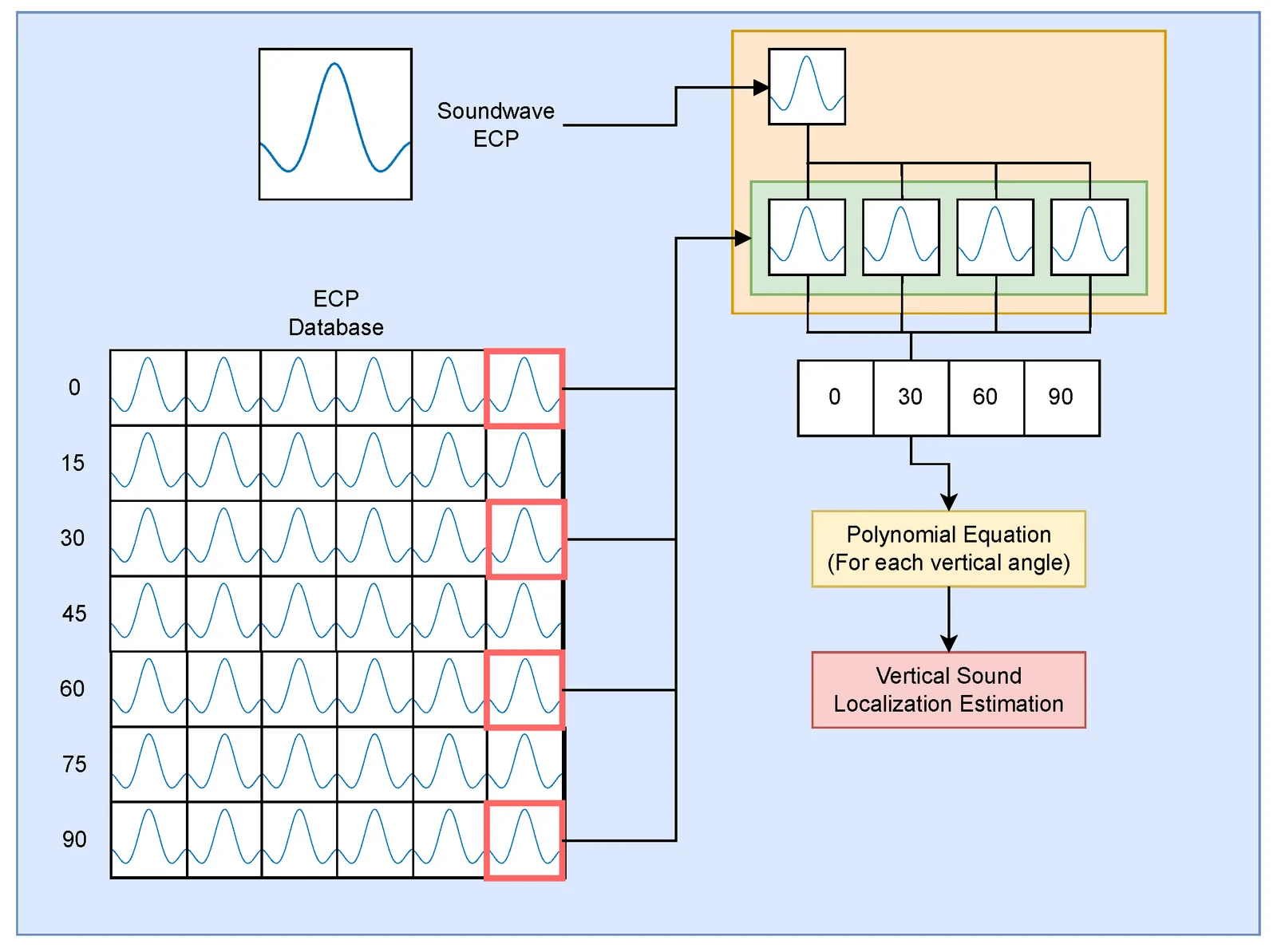
This system only uses the sound from one ear to determine the vertical angle of the unknown sound direction.

By the Cauchy–Schwarz inequality, $S_{j}$ has a theoretical range of [−1,1]. The scores in the present experiments are positive and therefore appear between 0 and 1 in Figure 13; they were not produced by a separate rescaling to [0,1]. Maximizing over *q* permits a relative displacement between the test and template ECPs. The score therefore measures similarity of the complete profile shape and its relative lag structure, rather than independently comparing a list of estimated physical gains and delays.

The available implementation calculates $S_{j}$ for seven recorded directions: $0^{\circ }$, $15^{\circ }$, $30^{\circ }$, $45^{\circ }$, $60^{\circ }$, $75^{\circ }$, and $90^{\circ }$. The subsequent continuous-angle estimation stage retains the four anchor profiles at $0^{\circ }$, $30^{\circ }$, $60^{\circ }$, and $90^{\circ }$ and applies the pre-calibrated polynomial mapping associated with the selected neighboring anchors. This separates the ECP similarity calculation from the later polynomial interpolation. Figure 13 summarizes the one-microphone localization pipeline.

**Figure 14:**
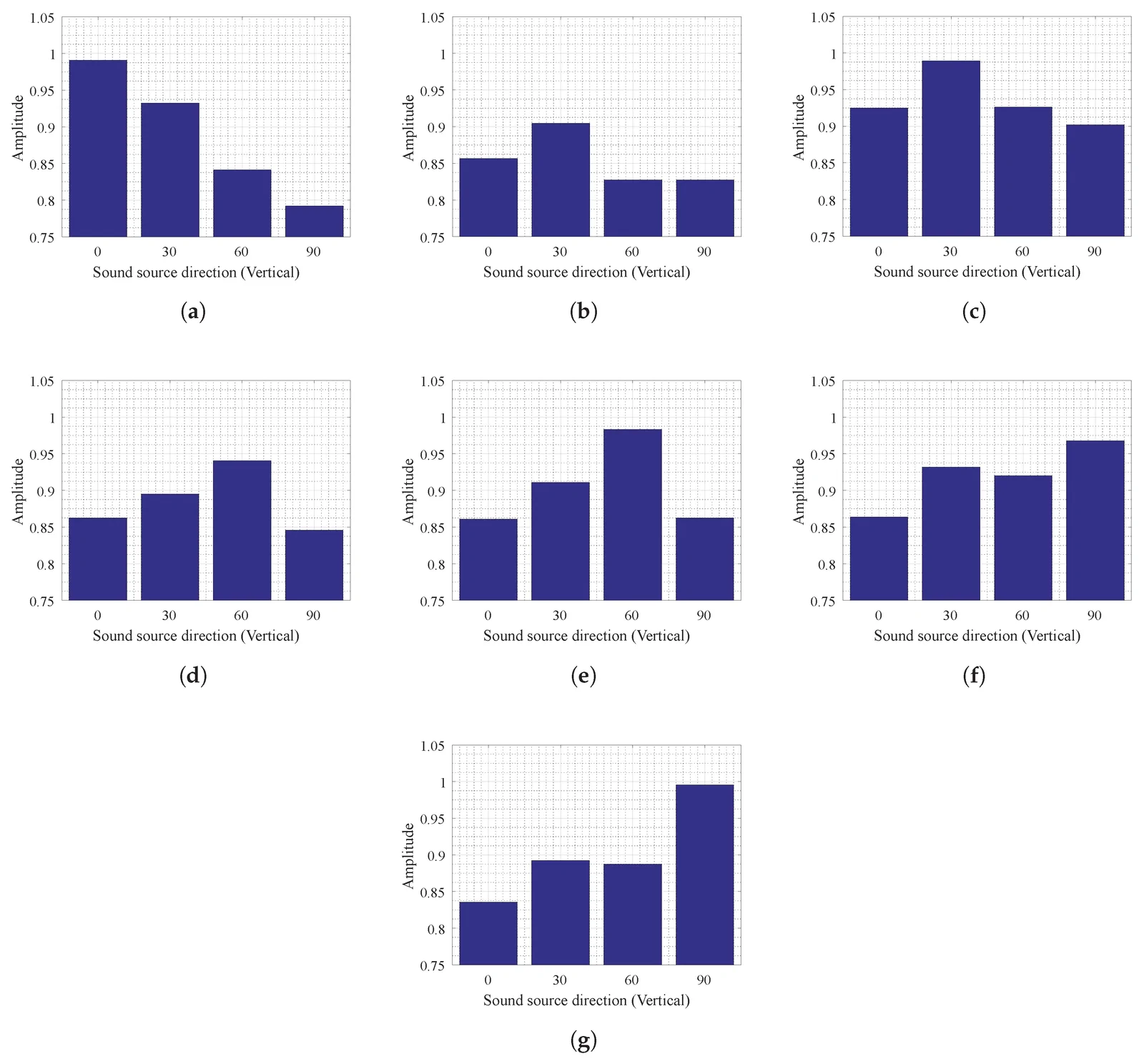
Maximum normalized cross-correlation coefficients between each test ECP and the stored ECPs: (**a**) $0^{\circ }$, (**b**) $15^{\circ }$, (**c**) $30^{\circ }$, (**d**) $45^{\circ }$, (**e**) $60^{\circ }$, (**f**) $75^{\circ }$, and (**g**) $90^{\circ }$.

The Echo Characteristic Profiles in the database are used to determine the unknown vertical direction by evaluating Equation (7) for each stored profile. Figure 14 shows the resulting maximum normalized cross-correlation coefficients.

The largest and second-largest anchor-profile scores identify the neighboring anchor interval used by the polynomial stage. For example, if the strongest anchor similarities correspond to $0^{\circ }$ and $30^{\circ }$, the associated pre-calibrated mapping estimates an angle within that interval. The polynomial mappings are fitted from the calibration recordings; they are applied after, and are mathematically distinct from, the normalized cross-correlation score in Equation (7).

### 2.6. Computational Complexity and Comparison Scope

Let *N* denote the number of samples in the selected waveform window, *L* the number of retained ECP lags, *M* the number of stored ECP templates, and *P* the number of polynomial coefficients evaluated for the selected interval. Computing only the required *L* lagged self-correlation values directly requires O(NL) operations. A direct full cross-correlation of two length-*L* profiles requires $O(L^{2})$ operations; comparison with all *M* profiles therefore requires $O(ML^{2})$ operations and O(ML) template storage. The final polynomial evaluation requires O(P) operations. FFT-based correlation can reduce the correlation stages to approximately O(NlogN+MLlogL), although the actual runtime depends on the implementation, signal length, hardware, and library routines.

Table 1 provides an architectural comparison rather than an accuracy or runtime benchmark. HRTF-, microphone-array-, and binaural-cue methods cover broad families with different sensor counts, representations, estimators, and acceleration strategies; a single generic asymptotic expression would therefore not constitute a fair performance comparison. A controlled benchmark must use the same recordings, angle set, hardware, error metrics, and runtime environment. Such a matched experiment was not conducted in the present study and remains necessary future work.

## 3. Results

### 3.1. Vertical Localization Results

The Echo Characteristic Profiles will determine the polynomial equation that will be used to calculate the sound source direction. We have a total of six polynomial equations that represent each of the sound source directions. Each of the sound source directions will be calculated using one of the six polynomial equations. The result for each of the sound source directions using the sampling rate of 192 kbps is shown in Table 2.

The overall Average Angular Error is 4.86 degrees, and the overall RMSE is 6.90. But the results show that the angles that also have the polynomial equation associated with them at the exact angle have low angular error. However, the sound directions that do not have the polynomial equation associated with them show higher angular error. Especially the 75-degree angle, which has the highest angular error. This might be because of the nature of the human ear, which might not be able to determine the sound direction from this particular angle.

Using the same method, we have also analyzed and determined the sound source direction when using the sampling rate of 48 kbps. The result is shown in Table 3.

The result of the sound with a sampling rate of 48 kbps has shown no significant difference than the sound with a sampling rate of 192 kbps. The overall Average Angular Error is 4.76, and the overall RMSE is 5.84. This result confirms that we do not need the sound resolution to be very high to determine the sound source direction.

**Figure 15:**
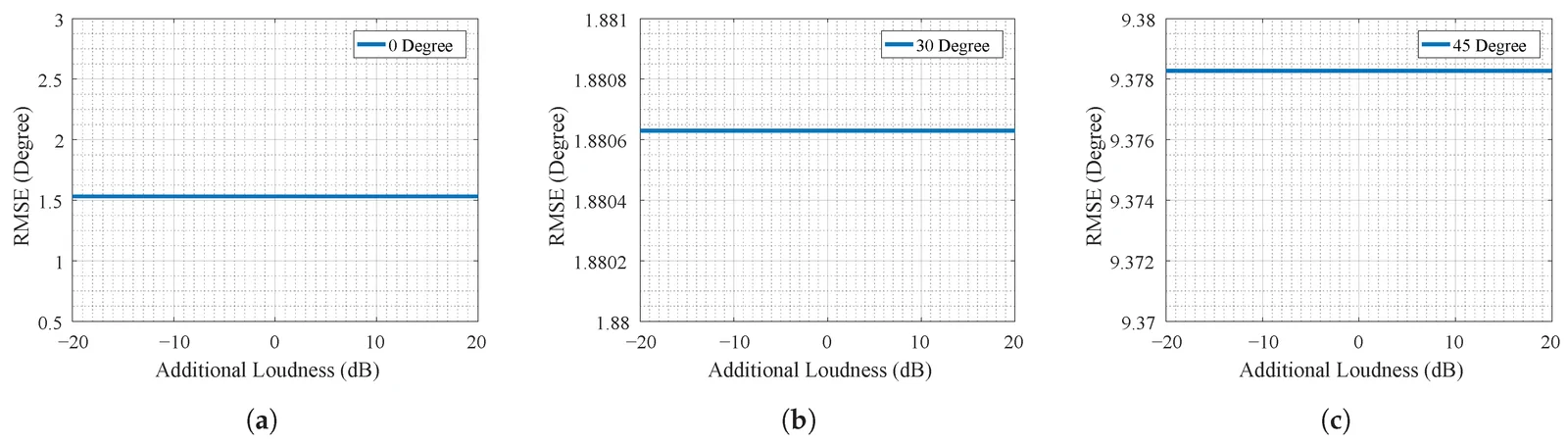
The result from the sound wave that has been modified for increasing/decreasing in loudness before using our system to localize the sound in each of the vertical planes: (**a**) 0 degrees, (**b**) 30 degrees, and (**c**) 45 degrees.

We have tested the different loudness levels of the signal. The loudness of our sample is between 66 and 68 dB. By adjusting the loudness of the signal from −20 dB to +20 dB, we have tested how loudness has an impact on our sound source direction determination. The results are shown in Figure 15.

The results show that each of the loudness levels that we adjusted from the sound wave has almost no impact on the overall result of our system. The sound source direction determination is almost the same for each loudness level that we adjusted. So we can say that the loudness of the signal has no impact on our system.

**Figure 16:**
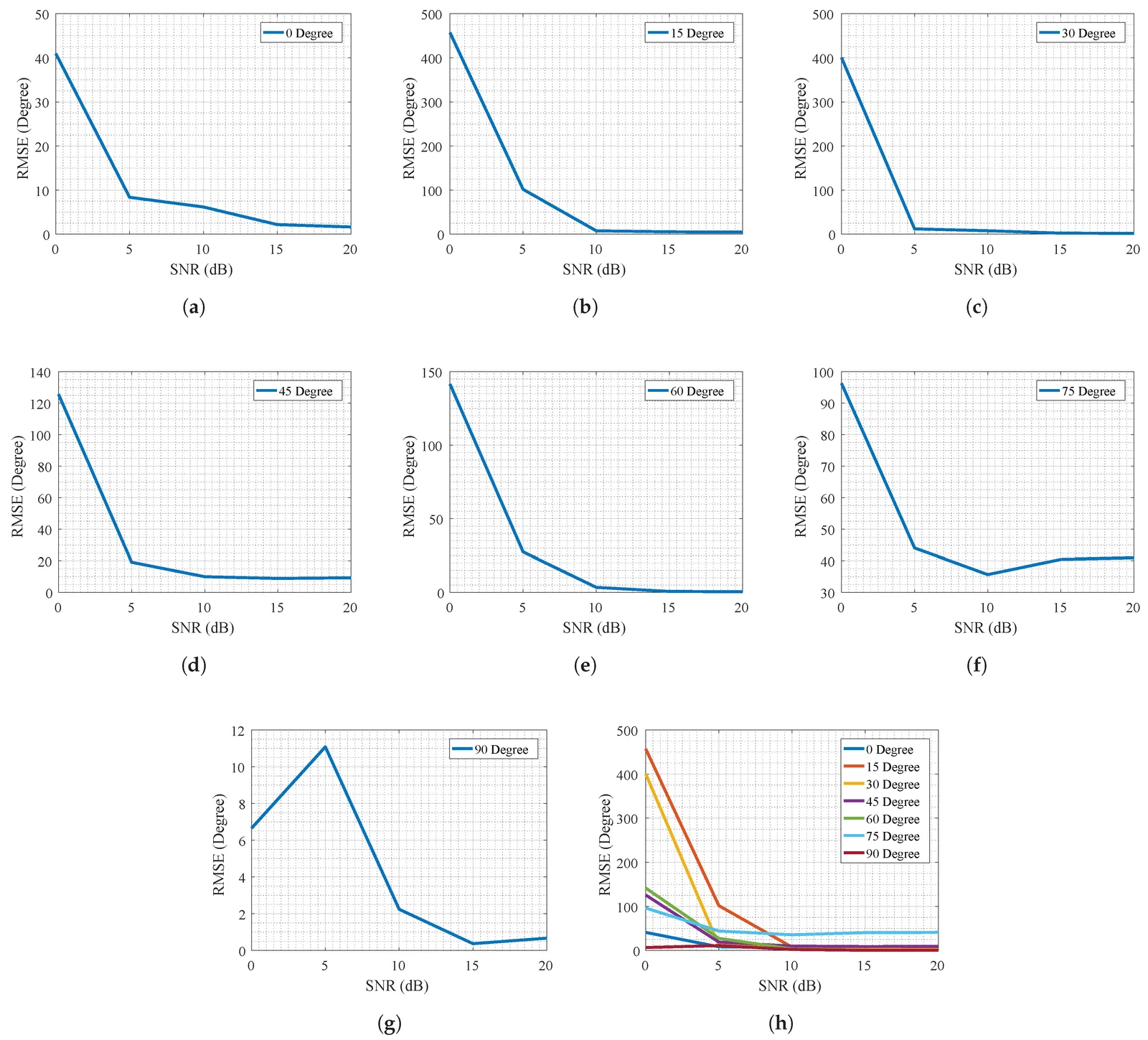
The results from the sound wave that has been modified by increasing/decreasing the SNR before using our system to localize the sound in each of the vertical planes: (**a**) 0 degrees, (**b**) 15 degrees, (**c**) 30 degrees, (**d**) 45 degrees, (**e**) 60 degrees, (**f**) 75 degrees, (**g**) 90 degrees, and (**h**) all vertical degrees.

We also tested the impact of each of the SNRs on our system. We adjusted the SNR from 0 dB to 20 dB, thus resulting in different SNR signals for each sound wave from each vertical direction. The results are shown in Figure 16.

The results show that most of the vertical sound source directions can still be used to determine an accurate sound source with an SNR of 5 dB. However, the system falls short at an SNR of 0 dB, most likely because it cannot detect the verbality of the sound. As the noise covers almost all of the sound completely. Thus, the system cannot determine the beginning of the sound and cannot determine an accurate direction of the sound source.

Coincidentally, the RMSE of the sound direction from 75 degree is also hugely different from the base result. As we showed, the base result of the sound direction that comes from 75 degrees already has the highest RMSE. Thus, when adding the noise, the result also showed a very high RMSE due to the noise obstructing the sound. Thus resulting in additional error in the system.

### 3.2. Comparison with Other Methods

Recent three-dimensional sound-localization studies have used distributed microphone arrays to improve spatial coverage and accuracy [26,31,34]. In the study considered here, microphones were separated by approximately 1 m and the complete array occupied an area of approximately 6 m. The reported mean localization error along the vertical (*Z*) axis was 0.4125 m. Because this quantity is a position error rather than an angular error, it cannot be compared directly with the elevation error reported for the proposed method.

To provide contextual scale only, we express the reported vertical-position error as an approximate elevation-angle error using the measurement geometry described in that study. The drone was evaluated at horizontal positions separated by approximately 2 m, with a fixed height of Z=5 m and a maximum range below 10 m. For each horizontal position, the horizontal distance from the array is calculated using Equation (9).

(9) $$D=\sqrt{\left(B^{2}+C^{2}\right)}$$

where *D* is the horizontal distance between the array and the drone, and *B* and *C* are its horizontal coordinates. The nominal elevation angle is then calculated from the fixed height *Z* and horizontal distance *D* using Equation (10).

(10) $$D_{a}=\arctan\left(\frac{Z}{D}\right)$$

where $D_{a}$ is the elevation angle, *Z* is the drone height, and *D* is defined in Equation (9). For example, at B=C=4 m, the nominal elevation angle is $41.47^{\circ }$. For a reported vertical error ΔZ, the corresponding local angular deviation is calculated as $\left|\arctan((Z+\Delta Z)/D)-\arctan(Z/D)\right|$. The resulting value is geometry-dependent and is therefore reported in Table 4 only as an approximate contextual conversion, not as a matched accuracy result.

Head-Related Transfer Function (HRTF) methods are also widely used for three-dimensional sound localization. However, the cited HRTF results jointly assess horizontal and vertical localization and use different ear models, recordings, estimators, and evaluation protocols. They are consequently not directly comparable with the present elevation-only experiment. Table 4 is therefore included as a descriptive reference: it summarizes approximate physical scale, sensor count, and reported or converted angular errors for selected methods, without supporting a claim of superior accuracy or runtime for the proposed method.

On closer examination of the other studies [26,31,34], most report their results using average angular error. In [26], a drone-localization system uses a microphone array. The system is large (approximately 6 m) and uses a total of eight microphones. The results were also reported in meters; therefore, we used the method described above to calculate the average angular error. The average angular error is low at 2.6 degrees, and the RMSE is 2.62. The experiment in [31], which uses the HRTF and CNN, adopts a different data-collection approach to localize sound in both the horizontal and vertical planes. It uses two microphones for each ear to localize the sound source. Its reported accuracy is high at 89%, and its best CNN configuration had an average angular error of 1.2 degrees. However, its SNR is reported only in the horizontal plane; therefore, it cannot be used for comparison with our vertical-localization system. The experiment in [34] uses the HRTF and DNNs to localize sound in both the horizontal and vertical planes. It reports accuracy and average angular error. Its best DNN setup had an accuracy of 60.43%, because the system tolerance was set to 0 degrees and the angular error started at 5 degrees with an increment of 5 degrees. As a result, its reported average angular error is very low at 2.6 degrees.

Although we cannot compare all the metrics reported in our results with other studies, Table 4 presents the metrics and results that can be compared with other studies.

## 4. Discussion

The results support the feasibility of using an angle-calibrated time-domain ECP to estimate vertical sound direction with the evaluated artificial ear. However, the present experiment does not establish lower runtime or higher accuracy than HRTF, microphone-array, or binaural localization methods because matched baseline experiments were not conducted. The analytical cost of the implemented stages is stated in Section 2.6, while comparative accuracy and measured runtime remain open evaluation requirements. The proposed method also requires ear-specific calibration: different ear shapes, and the left and right ears of the same model, may produce different ECPs and should therefore use separately calibrated profile sets.

We did an analysis on the SNR impact on the system. The result showed that SNR has some impact on the accuracy of the system. However, as long as the system can detect the main sound, it should still be used to calculate an approximate direction of the sound in the vertical plane. An SNR of 0 shows a much larger error in the system. While an SNR of 5 and higher shows that the system can determine the vertical direction close to the raw sound. This shows the system’s limitation in noise tolerance, as the system output of 0 SNR shows out of bound results. We also analyzed the sound when we adjusted the loudness. The result showed that the loudness of the sound has little to no impact on the accuracy of the system.

The current implementation performs lagged self-correlation and template cross-correlation directly on time-domain profiles. This design avoids an explicit frequency-domain feature representation, but that fact alone does not demonstrate lower runtime or energy consumption than other localization algorithms. Deployment claims require measured comparisons on common hardware and data. The present complexity analysis therefore characterizes only the proposed pipeline and does not assert computational superiority.

## 5. Conclusions

This experiment shows that a calibrated ECP derived from one microphone in an artificial ear can be used to estimate sound-source direction in the evaluated vertical plane. The revised formulation describes the received signal as a discrete angle-dependent multipath process and defines the ECP and template score using normalized lagged self-correlation and cross-correlation. The reported error values apply to the present ear, recordings, and calibration procedure. Claims of generalization to other sounds or ear geometries, and claims of lower computational cost than conventional HRTF or array methods, require controlled comparative experiments and are left for future work.

## Figures and Tables

**Table 1:** Architectural scope of the proposed ECP approach and commonly used localization families. This table is not an experimental performance comparison.

Method Family	Recording Setup	Representation and Calibration
Proposed ECP	One microphone in an artificial ear	Time-domain ECP from normalized lagged self-correlation; angle templates and polynomial calibration
HRTF matching	Usually two channels	Direction-dependent transfer functions and a subject- or device-specific HRTF database
Microphone array	Multiple microphones	Inter-sensor delays, beamforming, or spatial spectra; array geometry and calibration
Binaural ITD/IID	Two ear channels	Interaural timing and/or level cues; binaural geometry and cue model

**Table 2:** The result of the experiment showing the Angular Answer, Angular Error, and RMSE of the tested sound from each vertical angle. Using sampling rate of 192 kbps.

Actual Angle	Average Angular Answer	Average Angular Error	RMSE
0	1.70	1.70	1.7607
15	20.08	5.08	5.6298
30	30.23	1.75	1.7605
45	56.09	11.09	11.1186
60	59.33	0.67	0.6768
75	88.08	13.08	13.0801
90	90.68	0.68	0.6786

**Table 3:** The result of the experiment showing the Angular Answer, Angular Error, and RMSE of the tested sound from each vertical angle using sampling rate of 48 kbps.

Actual Angle	Average Angular Answer	Average Angular Error	RMSE
0	0.50	1.10	1.3595
15	18.33	3.33	3.4220
30	32.29	2.29	2.2977
45	38.18	12.35	19.4313
60	61.16	1.16	1.1702
75	87.61	12.61	12.6134
90	90.15	0.51	0.5515

**Table 4:** Contextual comparison of selected sound-localization methods. Mean estimated angle and RMSE are included where reported; “not reported” denotes that no directly comparable value was available. Values were obtained under different experimental conditions and do not constitute matched accuracy or runtime benchmark.

Method	Approximate System Size	Microphones Used	Mean Estimated Angle	Reported or Converted Angular Error	RMSE
Proposed method	Approximately 0.4 m	1	Angle-specific ^1^	4.86°	6.90
HRTF + DNN [34]	Approximately 0.3 m	2	Not reported	2.60° ^2^	Not reported
HRTF + CNN [31]	Approximately 0.3 m	2	Not reported	1.20° and 24.57° ^3^	Not reported
Microphone array [26]	Approximately 6 m	8	Not reported	2.60° ^4^	2.62 ^4^

^1^ Mean estimated angles are reported separately for each tested elevation in Table 2 and Table 3. ^2^ Result reported jointly for horizontal and vertical localization. ^3^ Results reported for the best and worst configurations, jointly across horizontal and vertical localization. ^4^ Approximate value converted from the reported vertical-position error using the geometry described in the cited study.

## Data Availability

The data are available from the corresponding author upon reasonable request.

## Abbreviations

The following abbreviations are used in this manuscript:

SSL	Sound Source Localization
HRTF	Head-Related Transfer Function
TDOA	Time Difference of Arrival
IID	Interaural Intensity Difference
ITD	Interaural Time Difference
ECP	Echo Characteristic Profile
SNR	Signal-to-Noise Ratio
RMSE	Root Mean Square Error
FFT	Fast Fourier Transform
ML	Machine Learning
CNN	Convolutional Neural Network
